# Chronic Pancreatitis: The True Pathogenic Culprit within the *SPINK1* N34S-Containing Haplotype Is No Longer at Large

**DOI:** 10.3390/genes12111683

**Published:** 2021-10-23

**Authors:** Na Pu, Emmanuelle Masson, David N. Cooper, Emmanuelle Génin, Claude Férec, Jian-Min Chen

**Affiliations:** 1 Inserm, Univ Brest, EFS, UMR 1078, GGB, F-29200 Brest, France; punayeah@163.com (N.P.); emmanuelle.masson@univ-brest.fr (E.M.); emmanuelle.genin@inserm.fr (E.G.); claude.ferec@univ-brest.fr (C.F.); 2 Department of Critical Care Medicine, Research Institute of General Surgery, Jinling Hospital, Medical School of Nanjing University, Nanjing 210016, China; 3 Service de Génétique Médicale et de Biologie de la Reproduction, CHRU Brest, F-29200 Brest, France; 4 Institute of Medical Genetics, School of Medicine, Cardiff University, Cardiff CF14 4XN, UK; CooperDN@cardiff.ac.uk

**Keywords:** chronic pancreatitis, enhancer, linkage disequilibrium, regulatory variant, *SPINK1* gene

## Abstract

A diverse range of loss-of-function variants in the *SPINK1* gene (encoding pancreatic secretory trypsin inhibitor) has been identified in patients with chronic pancreatitis (CP). The haplotype harboring the *SPINK1* c.101A>G (p.Asn34Ser or N34S) variant (rs17107315:T>C) is one of the most important heritable risk factors for CP as a consequence of its relatively high prevalence worldwide (population allele frequency ≈ 1%) and its considerable effect size (odds ratio ≈ 11). The causal variant responsible for this haplotype has been intensively investigated over the past two decades. The different hypotheses tested addressed whether the N34S missense variant has a direct impact on enzyme structure and function, whether c.101A>G could affect pre-mRNA splicing or mRNA stability, and whether another variant in linkage disequilibrium with c.101A>G might be responsible for the observed association with CP. Having reviewed the currently available genetic and experimental data, we conclude that c.-4141G>T (rs142703147:C>A), which disrupts a PTF1L-binding site within an evolutionarily conserved HNF1A-PTF1L *cis*-regulatory module located ∼4 kb upstream of the *SPINK1* promoter, can be designated as the causal variant beyond reasonable doubt. This case illustrates the difficulties inherent in determining the identity of the causal variant underlying an initially identified disease association.

Chronic pancreatitis (CP) is a complex disease that can be caused by genetic and/or environmental factors ([1] and references therein). Genetic studies over the past 25 years have served to identify a trypsin-dependent pathway that is central to our understanding of the etiology of CP [2]. The recognition of this pathway emerged from the identification and characterization of gain-of-function variants in *PRSS1* (encoding cationic trypsinogen; MIM# 276000) and loss-of-function variants in *SPINK1* (encoding pancreatic secretory trypsin inhibitor; MIM# 167790) and *CTRC* (encoding chymotrypsin C (MIM# 601405), which specifically degrades all human trypsinogen/trypsin isoforms) in patients with the disease ([3,4,5] and references therein) (Figure 1).

A diverse range of variants in the *SPINK1* gene has been reported in the literature [3,4]. Whilst the pathogenic relevance of large deletions, splice-site variants and nonsense variants is usually self-evident, that of other types of lesion, particularly missense variants, often has to be determined by in-depth functional analysis. In this regard, a missense variant in the *SPINK1* gene, p.Asn34Ser or simply N34S (c.101A>G; rs17107315:T>C) [6], represents one of the most important CP-associated heritable risk factors owing to its relatively high prevalence (an allele frequency of 0.009028 according to gnomAD v2.1.1 [7]) and its considerable effect size (odds ratio (OR) = 10.90; 95% confidence interval 7.56–15.72) in accordance with a recent meta-analysis [8]) worldwide. However, whether or not the N34S missense substitution per se represents the underlying pathogenic variant that predisposes to CP has been the subject of continued research interest over the past two decades. The clarification of this issue has not only considerable biological interest but also potential diagnostic and therapeutic value.

Witt and colleagues initially postulated that N34S, which is located near the reactive site of SPINK1 (Lys41-Ile42), might impair the enzyme’s inhibitory activity on prematurely activated trypsin within the pancreas, thereby conferring predisposition to CP [6]. However, functional analyses of the wild-type and N34S mutant SPINK1 enzymes expressed in *Saccharomyces cerevisiae* [9], Chinese hamster ovary cells [10], and human embryonic kidney 293T (HEK293T) cells [11] failed to identify any measurable effect of the N34S variant on the expression, secretion or trypsin inhibitory activity of SPINK1. An alternative hypothesis that either c.101A>G or one of its four *cis*-linked intronic variants may affect pre-mRNA splicing or mRNA stability [12] also failed to garner support from experiments employing cell culture-based minigene [13] and full-length gene [14,15] splicing assays. Herein, it should be emphasized that in our full-length gene splicing assay [14,15], it is the *SPINK1* genomic sequence extending from the translation initiation codon to the translation termination codon that was inserted into the expression vector. In other words, a possible effect of c.101A>G and its four *cis*-linked intronic variants on pre-mRNA splicing and/or mRNA stability was simultaneously analyzed in the gene’s natural genomic sequence context as far as the entire coding and intronic sequences are concerned. Employing both qualitative and quantitative reverse transcription-PCR (RT-PCR) analyses, we did not observe any measurable effect of the c.101A>G or its four *cis*-linked intronic variants on pre-mRNA splicing and/or mRNA stability [14,15]. Moreover, RT-PCR analysis of total RNA prepared from pancreatic tissue of two c.101A>G (N34S) homozygotes yielded only wild-type transcripts [16]. However, expression levels of *SPINK1* mRNA were not subjected to quantitative analysis in this latter study. Additionally, no significant single-tissue expression quantitative trait loci (eQTLs) for *SPINK1* in human pancreatic tissue are listed in the Genotype-Tissue Expression (GTEx) database [17].

These negative findings stimulated us to embark upon a new hypothesis-driven approach that sought a causal regulatory variant in linkage disequilibrium (LD) with c.101A>G (N34S). Employing an LD threshold of *r*^2^ ≥ 0.40 and querying the 1000 Genomes Project Phase 1 data in the context of the European population by means of HaploReg v4.1, we identified a total of 25 single nucleotide polymorphisms in strong LD (*r*^2^ values ranging from 0.87 to 1) with c.101A>G in the region spanning 20 kb 3’ of *SPINK1* to 18 kb 5’ of *SPINK1* (Figure 2).

Of the 25 LD variants only one, rs142703147:C>A (c.-4141G>T relative to the A of the translation initiation codon of *SPINK1* demarcated as +1 [19]), was found to be both located within an evolutionarily conserved region and one of the three most accessible chromatin regions in pancreatic tissue. The three most accessible chromatin regions each harbor a putative HNF1A−PTF1L *cis*-regulatory module (CRM) (a CRM, usually 100–1000 base-pairs in length, contains several transcription factor binding sites, and provides the structural basis for coordinating the action of the corresponding transcription factors required for the temporal and spatial expression of neighboring genes [20]). Both HNF1A and PTF1L are basic components of the exocrine pancreas-specific transcriptional network for digestive function and pancreatic acinar cell homeostasis [21,22,23]. Importantly, c.-4141G>T occurs within one of these CRMs and is predicted to disrupt the corresponding PTF1L-binding site. Co-transfection transactivation experiments [18] have demonstrated that this variant leads to reduced gene expression. Contemporaneously, Kereszturi and Sahin-Tóth reported that two pancreatic cancer cell lines heterozygous for the *SPINK1* N34S haplotype exhibited reduced expression of the variant allele and suggested that c.-4141G>T might be a candidate causal variant [24].

Taken together, the findings from the aforementioned studies, and particularly the two from 2017 [18,24], suggested that c.-4141G>T was likely to be the causal variant underlying the association of the *SPINK1* N34S haplotype with CP. This notwithstanding, three recent studies have claimed that the pathogenic mechanism underlying the *SPINK1* N34S haplotype is still unknown and have therefore continued to search for a possible direct effect of the N34S missense variant on *SPINK1* structure and function. Two of these studies involved purely hypothetical simulation or modeling [25,26]. The other explored the possible impact of physiological stress factors (e.g., altered ion concentrations, temperature shifts and environmental pH) on the structure and trypsin inhibitory function of SPINK1 (and its N34S variant) using biophysical methods, but did not obtain any significant findings [27]. Herein, it is pertinent to mention a recent study from the Sahin-Tóth laboratory [28] that pointed out two serious shortcomings of the two early N34S-SPINK1 binding studies [9,11]. First, the methodology used was semiquantitative at best. Second, the use of either nonsulfated recombinant human cationic trypsin, bovine trypsin or a commercial human trypsin of unspecified nature in the binding assays could have impacted the functional relevance of the binding assays given that human trypsins are invariably sulfated on Tyr154 [29,30]. Using authentic sulfated human trypsins and more robust experimental conditions, Sahin-Tóth and colleagues provided conclusive evidence that N34S has no impact on trypsin inhibition [28].

Finally, it should be noted that the c.-4141G>T functional enhancer variant is in perfect LD with c.101A>G (N34S) in the French population (*r*^2^ = 1; 548 chronic patients and 562 controls genotyped) as well as in the European population of the 1000 Genomes Project [31] but not in the Chinese population (*r*^2^ = 0.80; 1104 patients and 1196 controls genotyped) or the Indian population (*r*^2^ = 0.59; 347 patients and 264 controls genotyped) [18]. Conditional analyses performed at the time suggested that both variants influenced disease risk [18]. With hindsight, this latter observation should be interpreted with a considerable degree of caution. Our main concern lies with the data obtained from the Indian population for which the lowest *r*^2^ was observed. Only in the Indian population was c.101A>G (N34S) found to have a higher odds ratio (OR) for CP than c.-4141G>T (15.12 versus 14.82). By contrast, in the Chinese population, c.-4141G>T had a higher OR than c.101A>G (6.13 versus 5.47) whilst the two variants had an equal OR in the French population where they are in complete LD. It should also be pointed out that (i) of the three populations studied, the Indian population had the smallest sample size [18] and (ii) the Indian population is well known to have a complex population substructure [32]. The constellation of these concerns suggests that the counterintuitive result obtained from the Indian population probably arose as a consequence of sampling error and/or population stratification. However, an alternative possibility could conceivably be the presence of an additional CP-predisposing variant in the Indian population that is in stronger LD with c.101A>G than with c.-4141G>T.

In summary, based upon the currently available genetic and experimental data, we conclude that c.-4141G>T represents the true culprit underlying the association of the *SPINK1* N34S haplotype with CP. In other words, the true culprit underlying the *SPINK1* N34S haplotype-disease association should no longer be thought to be at large [33]. This notwithstanding, one may still argue that further experiments are needed to unambiguously confirm causality. For example, discarded biopsy or autopsy tissue from N34S heterozygotes could, in principle, be analyzed with a view to showing that the N34S-containing allele is indeed associated with reduced *SPINK1* mRNA expression in vivo. Alternatively, given the availability of 2 cell lines heterozygous for both the c.101A>G (N34S) and c.-4141G>T variants, CRISPR/cas9 technology could be employed to generate isogenic cell lines carrying only the c.101A>G (N34S) variant or the c.-4141G>T variant. Subsequent comparison of *SPINK1* mRNA expression levels in these cell lines could then serve to strengthen the evidence for causality. Indeed, it is always better to provide additional experimental evidence if resources are available. However, in this particular case, the evidence supporting our contention that the c.-4141G>T variant is the true pathogenic variant is, we believe, beyond reasonable doubt.

The conclusion that c.-4141G>T is the true pathogenic culprit within the *SPINK1* N34S-containing haplotype has one immediate clinical implication. Genetic correction of the c.-4141G>T variant in the corresponding carriers could be explored as a patient-tailored therapy.

## Figures and Tables

**Figure 1:**
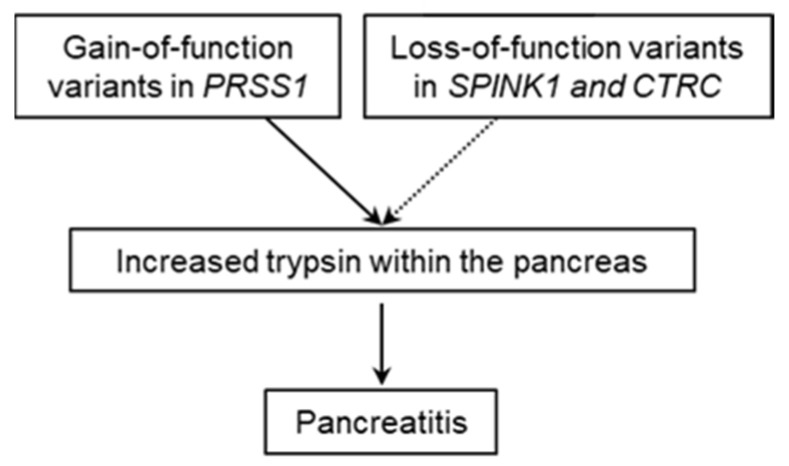
Schematic illustration of the trypsin-dependent pathway for chronic pancreatitis stemming from the identification of genetic variants in the *PRSS1*, *SPINK1,* and *CTRC* genes.

**Figure 2:**
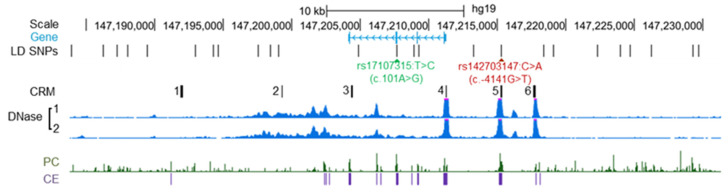
Illustration of the *SPINK1* locus, the locations of the *SPINK1* N34S (c.101A>G; rs17107315:T>C) and c.-4141G>T (rs142703147C>A) variants, and the spatial coincidence of c.-4141G>T with regulatory features. In the “Gene” panel, the four exons of the *SPINK* gene are denoted by vertical lines, with the arrows indicating the direction of transcription. LD SNPs, single nucleotide polymorphisms in strong linkage disequilibrium with *SPINK1* c.101A>G. It should be noted that only 24 of the 25 LD SNPs are shown in the Figure, the variant that was not shown, rs138251740A>G (located further downstream of chr5:147,230,000), is neither located within a putative PTF1L−HNF1A CRM nor within a region showing strong evolutionary conservation and high chromatin accessibility. CRM, *cis*-regulatory modules harboring binding sites for transcription factors HNF1A and PTF1L. DNase, DNase I-accessible DNA regions in the pancreatic tissues of two donors as determined by DNase-seq. PC and CE, Placental Mammal Conservation by PhastCons (PC) and Placental Mammal Conserved Elements (CE) were obtained from the UCSC Genome Browser. The Figure was adapted from our work [18] with permission (Copyright 2021 Wiley Periodicals LLC).
